# Differentiation and Generic Sentences

**DOI:** 10.1111/cogs.70057

**Published:** 2025-03-25

**Authors:** Patrick Rothermund, Roland Deutsch

**Affiliations:** ^1^ Department of Psychology II ‐ Social Psycholgy Julius‐Maximilians‐Universität Würzburg

**Keywords:** Generics, Generic sentences, Category learning, Differentiation, Stereotypes

## Abstract

Generic sentences such as “Birds lay eggs” are used frequently and effortlessly, but there is no simple quantitative rule that determines whether they are true or false. For instance, while “Birds lay eggs” is considered true, “Birds are female” is considered false, even though there are necessarily fewer birds that lay eggs than birds that are female. In this article, we adopt a cognitive perspective on genericity. Specifically, we draw on learning principles that predict asymmetries in the acquisition of category representations, which in turn might determine the acceptance of generic sentences. Our key hypotheses were that generics are more likely accepted when the attributes they refer to are distinctive (i.e., more prevalent in the category relative to comparison categories) and that this pattern is sensitive to the temporal order in which category information is acquired. We report three preregistered experiments to test these hypotheses. In all experiments, we employed a trait‐learning paradigm in which participants received information about exemplars of two fictitious kinds (human‐like sea creatures in Experiments 1–3, stones in Experiment 2) in sequential order. We manipulated the prevalence of attributes within kinds, as well as their status as being shared between kinds or distinctive for either the first‐ or second‐learned kind. As hypothesized, generic sentences were more likely accepted when referring to distinctive (vs. shared) attributes, but only for the second‐learned kind. We discuss implications for theories of generics as well as stereotype formation and representation.

## 1 Introduction

Generic sentences such as “Birds lay eggs” ascribe attributes to categories. Although they are regularly used and easy to comprehend, philosophers and linguists have struggled to identify conditions under which generic sentences are judged as true or false (Krifka et al., 1995; Leslie, 2008). Clearly, generic sentences allow exceptions, that is, an attribute need not apply to all category members for a sentence to be accepted. For instance, “Birds lay eggs” is widely considered true although only mature healthy female birds lay eggs. However, the number of exceptions that are tolerated seems to vary between different generics. For instance, “Birds are female” is widely considered false although there are necessarily more female birds than birds that lay eggs. Moreover, some generics are accepted despite very low prevalence levels (e.g., “Mosquitoes carry diseases” seems true although relatively few mosquitoes do), while other generics are rejected despite very high prevalence levels (e.g., “Germans are right‐handed” seems false although most Germans are).

### 1.1 A cognitive perspective on generics

Research efforts investigating the truth conditions of generic sentences typically adopt one of two perspectives, henceforth termed the semantic approach and the cognitive approach (Cohen, 2004; Lazaridou‐Chatzigoga, Katsos, & Stockall, 2015). The core assumption of the semantic approach is that the truth value of generic sentences is determined by linguistic rules (e.g., Carlson, 1982; Krifka et al., 1995; Pelletier & Asher, 1997). Scholars in this tradition seek to identify such rules in analytical fashion. Semantic analyses usually assume that generic sentences contain a covert operator akin to quantifiers like “all,” “some,” or “none.” For instance, generic sentences have been interpreted as meaning that “all normal” exemplars of the category possess the attribute (Pelletier & Asher, 1997). However, semantic analyses require additional constraints to account for otherwise paradoxical truth judgments. For instance, if generics would simply express that all normal exemplars of the category possess the attribute, it would be unclear why “Birds lay eggs” is widely considered true since it is not inherently abnormal for a bird to be male. Thus, Pelletier and Asher (1997) assume that the criteria need only apply to a relevant subset, for example, mature female birds (see also Lazaridou‐Chatzigoga, Stockall, & Katsos, 2019). The necessity of such constraints implies that generic sentences are somewhat more complex than ordinary quantified sentences, which express a straightforward statistical relation.

In contrast, the core assumption of the cognitive approach is that generic sentences express learned mental representations of categories (Gelman, 2010; Leslie, 2008). According to this view, category representations primarily consist of primitive propositional links that ascribe attributes to the category. These links vary in strength but do not encode quantitative information. For instance, “laying eggs” is strongly linked to “birds,” yet this link does not contain the information that it applies to less than half of all birds. Thus, the cognitive approach views generic sentences as “easy” in the sense that they express the default mode of linking attributes to categories (Leslie, 2008). This generics‐as‐default hypothesis received empirical support. For instance, multiple studies suggest that generics are acquired early in cognitive development. Specifically, young children (aged around 3 years old) start to master generics before being able to correctly apply quantifiers (Hollander, Gelman, & Star, 2002; Mannheim, Gelman, Escalante, Huayhua, & Puma, 2010; Tardif, Gelman, Fu, & Zhu, 2012). Other studies suggest that generic knowledge is more accessible than quantified knowledge. Specifically, people more often misremember quantified statements as generic than vice versa (Gelman, Leslie, Gelman, & Leslie, 2019; Leslie & Gelman, 2012). Under time pressure, quantified sentences tend to be judged as if they were generic sentences (Meyer, Gelman, & Stilwell, 2011). Taken together, these results suggest that information about categories is, by default, stored in generic rather than quantified form.

The cognitive approach allows novel predictions about generic sentences’ truth conditions. If generics reflect category representations, the key determinant of judging them as true or false should be how strongly attributes are linked to categories. Thus, factors that increase (decrease) the strength of category‐attribute links can be expected to also increase (decrease) the acceptance of corresponding generic sentences. One such factor is whether an attribute conveys danger or not. Dangerous information is privileged in attention, memory, and judgment (e.g., Baumeister, Bratslavsky, Finkenauer, & Vohs, 2001; Fiske, 1980; Pratto & John, 1991). Accordingly, generic sentences ascribing dangerous (vs. harmless) attributes to categories are more likely judged as true, even when statistical evidence is weak (Cimpian, Brandone, & Gelman, 2010; Leslie, 2017). For instance, “Sharks attack swimmers” is commonly judged as true, despite most sharks never encountering a swimmer. Another factor that affects the strength of category‐attribute links is the degree to which an attribute is essentialized, that is, how much it is considered a “natural product” of belonging to a category. Essentialized attributes are strongly embedded in causal beliefs, and more generally, overweighted in category‐related judgments (Ahn, Kim, Lassaline, & Dennis, 2000; Rehder & Hastie, 2001; Rothbart & Taylor, 1992). Correspondingly, in the realm of generic sentences, it has been argued that characteristic generics expressing a causal or “principled” connection between category and attribute are usually judged as true (Khemlani, Leslie, & Glucksberg, 2012; Leslie, 2008; Prasada, Khemlani, Leslie, & Glucksberg, 2013). This holds even when contradicting statistical information is provided (Gelman & Bloom, 2007). For instance, the acceptance of the generic “Dogs reproduce” seems invariant to the number of neutered pet dogs, likely because reproduction is seen as an inherent trait of animal species. In sum, factors such as the danger or essentialism of an attribute influence category representations as well as the acceptance of the corresponding generic sentence, which is in line with a cognitive view of generics.

### 1.2 Category differentiation

In this article, we aim to examine a factor beyond danger and essentialism that modulates how strongly attributes are integrated into category representations. Specifically, we propose that the extent to which an attribute is distinctive for a category influences the strength of the link between category and attribute in memory, and thus influences the acceptance of corresponding generic sentences. We conceptualize distinctiveness as the degree to which an attribute is predictive for a category relative to comparison categories (Cree, McNorgan, & McRae, 2006; Kruschke, 2003). Thus, distinctiveness is maximal when the attribute is present in a category but absent in comparison categories, as the attribute perfectly predicts the category in these cases. However, an attribute also qualifies as distinctive when merely being more prevalent in the category relative to alternative categories.

Whereas distinctiveness is a stimulus attribute, differentiation refers to the cognitive process of prioritizing distinctive information (Alves, Koch, & Unkelbach, 2018). Differentiation has been thoroughly examined as a general determinant of category formation in cognitive and social psychology (e.g., Alves et al., 2018; Davis & Love, 2010; Kruschke, 2003; Rothermund & Deutsch, 2024; Sherman et al., 2009). Seminal models of associative learning (Rescorla & Wagner, 1972) and category learning (Kruschke, 2003) predict that learning is driven by novel and diagnostic information. Indeed, distinctive features and exemplars are memorized better and categorized faster than nondistinctive ones (Cree et al., 2006; Davis & Love, 2010). For instance, the first attribute that comes to mind about zebras might be their stripes but hardly their legs. The tendency to focus on differences between concepts permeates basal cognitive functions such as visual processing (Lupyan, 2012). The mere act of providing category labels enhances visual discrimination between categories based on their distinctive attributes (Goldstone, 1994; Lupyan, 2008). Differentiation also shapes social perception, as groups are described and evaluated based on features and members that distinguish them from other groups (Alves et al., 2018; Ford & Stangor, 1992; Sherman et al., 2009; Woitzel & Alves, 2024). This can lead to polarized group judgments (Krueger & Rothbart, 1990; Rothermund & Deutsch, 2024). For instance, scientists might be characterized as nerdy and politicians as dull, but neither group as friendly, because friendliness is prevalent across groups but not differentiating between them (Unkelbach, Koch, & Alves, 2019).

In conclusion, distinctive attributes are prioritized in category formation, leading to the hypothesis that generic sentences about these attributes are more likely to be accepted than those about shared attributes. However, it should be clarified that we do not expect distinctiveness to directly influence the evaluation of generics during the moment of judgment. Rather, as discussed, distinctiveness strengthens category‐attribute links alongside other factors such as danger and essentialism. Some types of generics such as definitional generics (e.g., “Triangles have three sides”) or deeply essentialized generics (e.g., “Dogs reproduce”) are likely resistant to differentiation effects because they reflect inherent or structural properties of a category rather than learned associations. In contrast, generics that emerge from experience‐based category representations may be more susceptible to differentiation.

Initial empirical evidence confirms that generics about distinctive (vs. shared) attributes are more likely judged as true. Cimpian et al. (2010) investigated the acceptance of generic sentences about previously introduced fictitious animal kinds. Specifically, they presented statements about these kinds (e.g., “50% of lorches have purple feathers.”) and subsequently asked participants to judge the corresponding generic statement (“Lorches have purple feathers.”) as true or false. In addition to the prevalence of the attribute (the percentage of lorches that supposedly had purple feathers), its distinctiveness was manipulated. Specifically, some statements were followed up by a description that only the mentioned category possessed the attribute (e.g., “50% of lorches have distinctive purple feathers. No other animals on this island have wide, smooth feathers like these.”). Generic sentences in this condition were accepted more often than generic sentences in the control condition that described nondifferentiating attributes. In another study (Bian & Cimpian, 2021), similar results were found for both generics about categories as well as generics that generalized behavior to an individual.

In sum, two previous studies suggest that generic sentences about distinctive attributes are more likely endorsed than generic sentences about shared attributes (Bian & Cimpian, 2021; Cimpian et al., 2010). However, the interpretation of these results is limited because the experimental paradigms used in both studies confound distinctiveness with communicative emphasis. Specifically, in Cimpian et al. (2010), the descriptions of distinctive attributes were significantly longer than those of the shared attributes. Moreover, participants were asked to judge generics that explicitly highlighted distinctiveness (e.g., “Lorches have distinctive purple feathers.”, emphasis added), rendering them qualitatively different from the unmarked generics judged in the nondistinctive condition. In Bian and Cimpian (2021), the descriptions of distinctive attributes were followed up by the information “This behavior is extraordinary.”, while the descriptions of shared attributes were followed up by the information “This behavior is unremarkable.” Again, this communicates importance beyond distinctiveness itself. These limitations call for additional studies testing the mere influence of distinctiveness by manipulating it in a more subtle and ecologically valid way.

### 1.3 Differentiation and learning order

As described above, the cognitive account of generics predicts that under otherwise equal conditions, statements are more likely accepted when referring to distinctive (vs. shared) attributes, which is precisely the pattern observed in previous studies (Bian & Cimpian, 2021; Cimpian et al., 2010). However, while this aligns with a cognitive account of generics, other explanations of the effect are also possible. Some semantic analyses of genericity suggest that generic sentences simply mean that the prevalence of the attribute is higher in the mentioned category than in salient alternative categories (Cohen, 1999; van Rooij & Schulz, 2020). Furthermore, the distinctiveness effect might reflect the adherence to conversation principles. Specifically, the information that a category possesses an attribute that every other category also possesses might be considered trivial and thus redundant, and the communication of redundant information is usually avoided (Grice, 1975). In short, the prioritized acceptance of generics about distinctive attributes is not necessarily caused by specificities of mental representations but might instead reflect the application of semantic rules or conversation principles.

How to test the cognitive approach against these alternative explanations? We propose that the cognitive approach makes unique predictions when considering the temporal order of acquiring category information. This idea builds on the assumption that intercategory differentiation originates from category learning, where distinctive information is overweighted (Kruschke, 2003; Rescorla & Wagner, 1972). Obviously, this is only possible when distinctive information is recognized as such during that stage. Thus, whether an attribute is overweighted in category representations crucially depends on the presence of salient alternative categories during learning: only if comparison categories are salient, a novel category is differentiated from them based on distinctive attributes (Alves et al., 2018; Kruschke, 2003; Rothermund & Deutsch, 2024; Sherman et al., 2009; Woitzel & Alves, 2024). This implies that the learning order of multiple categories matters. When a category is learned first, no comparison standard exists, so its attributes are not recognized as distinctive. In contrast, when a second category is learned, the first category serves as a salient comparison, leading to stronger differentiation of the second category's distinctive attributes. For example, novel food categories such as plant‐based burgers are typically differentiated from conventional food categories such as meat‐based burgers, but not the other way around (Florack, Koch, Haasova, Kunz, & Alves, 2021). In the social domain, minorities and out‐groups typically constitute novel groups that are differentiated from their respective comparison groups (majorities and in‐groups), but not the other way around (Alves et al., 2018; Bergh & Lindskog, 2019).

In conclusion, the presence of distinctive attributes per se is not sufficient to induce differentiation. Differentiation originates from category learning, so distinctive attributes must be identifiable as such during this stage for the category to be differentiated from alternative categories. This applies to second‐learned but not first‐learned categories, a pattern that might be reflected in the judgment of generic sentences. Specifically, generic sentences about second‐learned categories might be more likely accepted if they ascribe a distinctive attribute to a category, whereas this effect might be attenuated or eliminated for first‐learned categories. Importantly, this asymmetry is uniquely compatible with a learning‐based cognitive account of generics. In contrast, neither semantic nor communicative principles predict such an interaction. Semantic rules hardly prescribe different truth conditions for first‐ and second‐learned categories. Communicative principles, on the other hand, might predict that the judgment of generics is sensitive to the salience of comparison categories in the current conversational context, but not to the salience of comparison categories in the prior stage of category learning (Grice, 1975; Tessler & Goodman, 2016).

### 1.4 The present research

Taking a cognitive perspective of generics, the present experiments tested two major hypotheses. First, we predicted that generic sentences are more likely judged as true when referring to distinctive (vs. shared) attributes. Although this distinctiveness effect has been observed in previous research (Bian & Cimpian, 2021; Cimpian et al., 2010), distinctiveness was confounded with communicative emphasis in these studies. Second, we tested the novel hypothesis that the effect is moderated by learning order. Specifically, generic sentences about distinctive (vs. shared) attributes might only be more likely accepted when describing second‐learned, but not first‐learned categories. This pattern is exclusively compatible with a learning‐based cognitive account of genericity.

We conducted three experiments to test these hypotheses. In all experiments, we employed an adapted version of the trait‐learning paradigm developed by Alves et al. (2018). In their study, participants imagined encountering members of two fictitious “alien tribes” and sequentially viewed individual tribe members, each represented by a drawing and a personality trait. Similarly, in our learning setup, participants encountered members of two fictitious kinds (sea creatures or stone kinds) in sequential order. Structurally, we deviated from the Alves et al. (2018) study by presenting eight (instead of six) exemplars per kind, and by presenting three (instead of one) attributes per exemplar. These modifications allowed us to distribute attributes to both kinds in line with our manipulations. In all experiments, the attributes varied in their prevalence within each kind and in whether they were exclusive to one kind (i.e., distinctive) or shared between both. After learning, we measured participants’ truth judgment of generic sentences that generalized these attributes to the kinds.

## 2 Experiment 1

### 2.1 Method

#### 2.1.1 Participants

The experiment was conducted as part of a student project under the supervision of the first author. In line with the preregistered protocol (https://aspredicted.org/m7sp‐d8s9.pdf), we collected data for 2 weeks, advertising the experiment on the recruiting platform of the university. Most participants were psychology students who earned course credit for participation. We collected complete datasets from 227 participants. Eight were excluded for failing attention check items, following the preregistered criterion. This resulted in a final sample of 219 participants (192 women, 26 men, 1 nonbinary; *M*_age_ = 22.2, *SD*_age_ = 6.3).

#### 2.1.2 Design

The experiment employed a 3 (prevalence: 25% vs. 50% vs. 75%) x 2 (distinctiveness: distinctive vs. shared) x 2 (position: first vs. second) design, all factors manipulated within subjects.

#### 2.1.3 Stimuli

We invented names for two fictitious sea creature kinds, the “Aquarids” and the “Neptunians.” A student created two drawings of sea creatures that were used to represent both kinds in the experiment. In both drawings, the creature was depicted as having a human‐like face and upper body, but jellyfish‐like tentacles instead of legs. To differentiate between the two kinds, one kind had yellow skin, while the other one had blue skin. The drawings are available on OSF. We generated nine attribute descriptions. Each description briefly introduces a human‐like personality trait of positive valence. For example, we used the following description for the trait creativity: “With his artistic talent, this Aquarid / Neptunian never ceases to amaze his friends. He has a lot of unusual ideas. He is very creative.” A list of all attribute descriptions is available on OSF. To implement the manipulations, all attribute descriptions were randomly distributed to nine roles for each participant (see Table 1), determining how prevalent the attributes were among both kinds. We also randomly assigned the kind names (Aquarids / Neptunians) to the first‐ and second‐learned kind, and randomly selected which drawing belonged to which kind.

**Table 1:** Attribute roles used in all experiments

Attribute role	Prevalence in first‐learned kind	Prevalence in second‐learned kind
DistinctiveFirst25	25%	0%
DistinctiveFirst50	50%	0%
DistinctiveFirst75	75%	0%
DistinctiveSecond25	0%	25%
DistinctiveSecond50	0%	50%
DistinctiveSecond75	0%	75%
Shared25	25%	25%
Shared50	50%	50%
Shared75	75%	75%

*Note*. To implement the manipulations, each attribute was randomly assigned to one of the nine roles for each participant. The attribute role determined whether it was distinctive for the first‐learned kind, distinctive for the second‐learned kind, or shared between kinds, and how prevalent the attribute was within the kind(s). For instance, the DistinctiveFirst25 attribute refers to the attribute that was distinctive for the first‐learned kind with a prevalence of 25%, thus possessed by 25% of the exemplars of the first‐learned kind but not possessed by any exemplar of the second‐learned kind.

#### 2.1.4 Procedure

Participants were linked to the online experiment that was implemented on the platform SoSciSurvey, where they first gave consent to participate. Then, they were instructed that the experiment required them to imagine a fictitious scenario. In a nutshell, this scenario describes that it is the year 2050 and that the participant is part of a research group that discovered the existence of two human‐like sea creature kinds, the Aquarids and the Neptunians. The full instruction text is available on OSF. After this instruction, a screen containing four attention check items was presented (e.g., “It is the year 2023,” which was false). When failing at least one item, participants were directed back to re‐read the scenario. On the next screen, participants were instructed to imagine that they were meeting eight exemplars of the first kind (Aquarids or Neptunians), that they were going to see one exemplar after another, and that their task was to form an accurate impression of both kinds. In line with these instructions, eight exemplars of the first kind were presented on separate screens that participants clicked through manually. Each exemplar consisted of the drawing selected for the kind and three of the attribute descriptions selected in accordance with the distribution explained previously. For example, the attribute description assigned to the DistinctiveFirst25 role was presented alongside two out of eight exemplars of the first kind. After seeing all exemplars of the first kind, participants were instructed that they were going to see eight exemplars of the second kind (Aquarids or Neptunians) and that their task was to form an accurate impression of the kind. Again, eight exemplars were presented sequentially, including the other drawing and three attributes each. After the learning phase, participants were asked to keep their impression of both kinds in mind and instructed that the next screen would display sentences about one of the two kinds (Aquarids or Neptunians) that they had to judge. The exact wording of the task was “Please indicate whether you would agree or disagree with these sentences based on your experiences!”. In line with these instructions, the next screen displayed generic sentences that ascribed an attribute to the kind (e.g., “Neptunians are creative.”). For each sentence, participants indicated whether they agreed with this sentence or not by clicking the respective button. We included generic sentences about all attributes that were indeed possessed by members of that kind, resulting in a total of six sentences. The order of sentences was randomized. In addition to the six sentences, an attention check item was mixed in (“This is an attention check. Please click ‘no’.”). Failing this item led to an exclusion of the participant's data. After responding to the generic sentences, the next screen displayed the same instructions for the other kind (Aquarids or Neptunians), and six generic sentences were presented on the next screen in an equivalent fashion. Whether participants first judged generic sentences about the first‐ or second‐learned category was chosen randomly. On the final screen, participants provided demographic information before being thanked and linked back to the acquisition platform.

#### 2.1.5 Dependent variables

The judgment of generic sentences (acceptance vs. rejection; binary) served as the only dependent variable.

### 2.2 Results

**Fig. 1:**
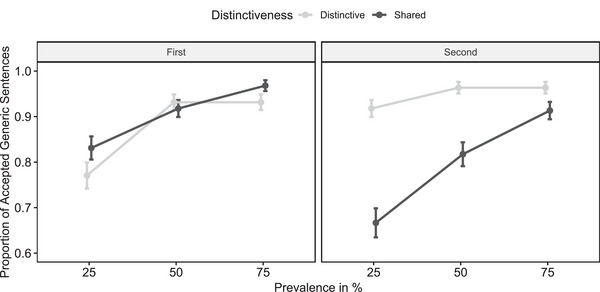
Study 1 results. *Note*. Proportion of accepted generic sentences as a function of prevalence, distinctiveness, and position (Study 1). Error bars represent the standard errors of means.

Data and analysis scripts are available on OSF. Descriptive results are summarized in Fig. 1. To analyze the acceptance of generic sentences, we employed a generalized mixed effects model using the glmer function in the R package lme4 (Bates, Mächler, Bolker, & Walker, 2015). Single judgments (1: accept; 0: reject) were predicted by prevalence (25% vs. 50% vs. 75%, as linear predictor), distinctiveness (distinctive vs. shared), and position (first‐learned vs. second‐learned kind) as well as their interactions as fixed effects. We further included participant intercepts as random effects. In line with our hypotheses, generic sentences about distinctive attributes were more likely accepted than generic sentences about shared attributes, indicated by a significant main effect of distinctiveness, *z* = 3.90, *p* < .001. As predicted, this effect was qualified by a significant interaction between distinctiveness and position, *z* = 6.14, *p* < .001. Contrast tests (two‐tailed) revealed that distinctive (vs. shared) attributes were more likely accepted only for the second‐learned category, *z* = 6.99, *p* < .001, but not for the first‐learned category, *z* = 1.61, *p* = .101. Furthermore, and in line with our hypotheses, the prevalence of an attribute significantly influenced the acceptance of the corresponding generic sentence, *z* = 8.50, *p* < .001.

### 2.3 Discussion

The results of Experiment 1 confirm our main predictions. Specifically, generic sentences about distinctive attributes were more likely accepted than generic sentences about shared attributes, but only for the category encountered second, not for the category encountered first. Furthermore, as expected, generic sentences were more likely accepted the more prevalent the generalized attribute was. Theoretical and practical implications of these results will be taken up in detail in the General Discussion.

We designed a second experiment to replicate and extend the findings of Experiment 1. Specifically, we tested whether the pattern of results generalizes to nonsocial categories. Therefore, we varied whether the scenario described fictitious sea creature kinds or fictitious stone kinds. Furthermore, in contrast to Experiment 1, we used physical properties instead of personality traits. Physical properties have the obvious advantage of being applicable to both sea creatures and stones. Moreover, they cut the potential influence of participants’ naïve personality theories to a minimum. Specifically, in Experiment 1, participants likely expected an a priori correlation between some personality traits. This might have inflated the overall acceptance of generic sentences (e.g., if Neptunians are creative, they might also be considered intelligent). In contrast, the physical property attributes chosen for Experiment 2 were not a priori related to each other.

From a theoretical point of view, the main question addressed in Experiment 2 was whether judgments of generic sentences differ between inanimate categories (stones) and social categories (sea creatures). Our prediction was that the key result of Experiment 1, that is, the interaction between distinctiveness and position, would be replicated across both category domains. Specifically, we expected that sentences referring to distinctive attributes were more likely accepted than sentences referring to shared attributes, but only for second‐learned categories, and that this pattern occurred for both sea creatures and stones. We did not see any theoretical reason to expect differences between domains. Differentiation has been observed in both social and nonsocial category learning (e.g., Sherman et al., 2009). Moreover, both social and nonsocial generics might reflect mental representations (e.g., Leslie, 2008). Nevertheless, it was important to test the generality of the phenomenon across domains.

Apart from distinctiveness, judgments of generics about inanimate and social categories might differ in other ways, which we examined as additional research questions. First, we considered the possibility that generics about stones (vs. sea creatures) are accepted more likely overall. For inanimate categories, people might require less evidence to generalize from single instances to the whole category because less variability within categories is expected. For example, learning that an individual stone is heavy might be generalized to the stone kind, while the information that an individual sea creature is heavy might be attributed to individual differences. Second, the category domain might influence the acceptance of generic sentences differently depending on the attributes’ prevalence. Specifically, generics about stones (vs. sea creatures) might be less likely accepted at low prevalence levels, but more likely at high prevalence levels. A similar interaction effect was found in a study by Cella, Marchak, Bianchi, and Gelman (2022) comparing the acceptance of generics about human and animal kinds. The authors concluded that perceivers tend to an “all‐or‐nothing” generalization scheme for nonhuman categories as they are perceived as rather homogeneous, while generalizations about human categories might be more variable.

## 3 Experiment 2

### 3.1 Method

#### 3.1.1 Participants

In line with the preregistered protocol (https://aspredicted.org/XVL_CGW), we recruited 250 participants via Prolific.^1^ Two participants were excluded for failing attention check items, following the preregistered criterion. This resulted in a final sample size of 248 (88 women, 156 men, 4 nonbinary; *M*_age_ = 33.5, *SD*_age_ = 10.8).

#### 3.1.2 Design

The experiment employed a 3 (prevalence: 25% vs. 50% vs. 75%) x 2 (distinctiveness: distinctive vs. shared) x 2 (position: first vs. second) x 2 (domain: creatures vs. stones) design. The factor domain was manipulated between‐subjects, all other factors were manipulated within‐subjects.

#### 3.1.3 Stimuli, procedure, and dependent variables

Stimuli, procedure, and dependent variables were structurally adopted from Experiment 1 but modified in several ways. First, we implemented the factor domain. Specifically, the fictitious scenario described newly discovered sea creature kinds for only half of the participants, while it described newly discovered stone kinds (“Aquarite Stones” and “Neptolite Stones”) for the other participants. We slightly modified the description of the fictitious scenario to make it equally suitable for both domains. The modified instruction is available on OSF. Second, we did not present drawings to depict the kinds anymore. Instead, before each exemplar, we presented the sentence “Information about the next exemplar will appear here soon!” for 2 s before the three attribute descriptions appeared automatically. Third, we generated nine novel attribute descriptions. Instead of the personality traits used in Experiment 1, we generated physical properties that were used for both stone kinds and sea creature kinds (e.g., “This Aquarid / Neptunian / Aquarite Stone / Neptolite Stone reflects a considerable amount of incident light.”). The full list of attributes is available on OSF. Finally, we exchanged the exact wording used to instruct the judgment of generic sentences to the following: “Based on the information you have just read, please indicate whether you agree or disagree with these sentences. Decide whether you think the sentences are an applicable description of the kind in general.”

### 3.2 Results

**Fig. 2:**
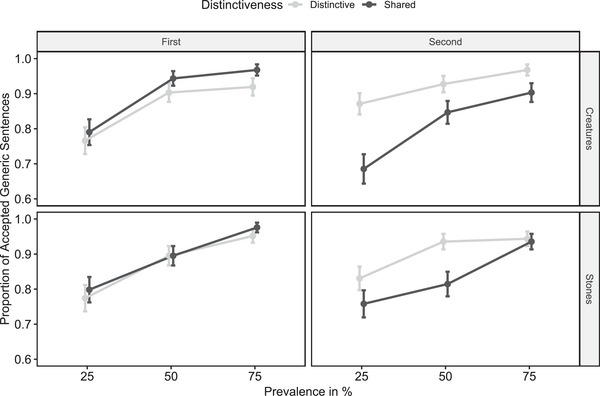
Study 2 results. *Note*. Proportion of accepted generic sentences as a function of prevalence, distinctiveness, position, and domain (Study 2). Error bars represent the standard errors of means.

Data and analysis scripts are available on OSF. Descriptive results are summarized in Fig. 2. Building on the analysis protocol of Experiment 1, we employed a generalized mixed effect model but further included domain (creatures vs. stones) and its interactions with all other predictors as fixed effects. Replicating the main result of Experiment 1, we observed a significant interaction between distinctiveness and position, *z* = 5.02, *p* < .001. Contrast tests (two‐tailed) revealed that distinctive (vs. shared) attributes were more likely accepted for the second‐learned category, *z* = 5.03, *p* < .001. In contrast, distinctive (vs. shared) attributes were less likely accepted for the first‐learned category, *z* = 2.22, *p* = .027. Overall, in contrast to Experiment 1, the main effect of distinctiveness did not reach significance, *z* = 1.78, *p* = .075. As in Experiment 1, the prevalence of an attribute significantly influenced the acceptance of the corresponding generic sentence, *z* = 10.96, *p* < .001. The factor domain did not influence the acceptance of generic sentences, neither as a main effect nor in interaction with any other factors (all *p*s ≥ .129).

### 3.3 Discussion

The main goal of Experiment 2 was to test the generality of Experiment 1 results across different attributes (Experiment 1: personality traits; Experiment 2: physical properties) and category domains (Experiments 1 and 2: sea creatures; Experiment 2: stone kinds). The key result of Experiment 1 was replicated. Specifically, generic sentences were more likely accepted when referring to distinctive (vs. shared) attributes to categories, but only for second‐learned categories. As expected, this pattern emerged uniformly for both sea creatures and stone kinds. In contrast to Experiment 1, generic sentences about distinctive (vs. shared) attributes were even less likely accepted for first‐learned categories, although this effect was smaller than the distinctiveness advantage for second‐learned categories. This unexpected effect might be due to a general memory advantage for shared (vs. distinctive) attributes as they were presented in both categories and thus twice as often. As in Experiment 1, generic sentences were more likely accepted at higher prevalence levels. As additional research questions, we investigated whether the acceptance of generic sentences about stone kinds and sea creatures differed overall, or differently depending on prevalence, as indicated by previous research (Cella et al., 2022). We did not observe these differences between category domains. Apparently, attributes are generalized to different types of categories in a similar fashion, although it cannot be ruled out that some category domains that were not examined are in some way extraordinary (e.g., human groups, Cella et al., 2022).

A perhaps surprising result of both experiments is the overall high acceptance of generic sentences. Across conditions, generic sentences were accepted as true by more than 60% of participants, even when ascribing attributes that were possessed by only 25% of exemplars in the category. On the one hand, this might be interpreted as evidence for a primitive mechanism of linking attributes to categories even in the absence of strong statistical contingencies, which is in line with a cognitive view of generics (Leslie, 2008; see also Sutherland, Cimpian, Leslie, & Gelman, 2015). On the other hand, the high acceptance rates might indicate that the experimental task invited a very liberal interpretation of generic sentences. Specifically, participants might have treated their task as a memory test and accepted generics whenever recalling at least one exemplar of the category that possessed the trait. We designed a third experiment to address this point. In Experiment 3, each generic sentence was preceded by a memory test item in which participants indicated whether they recalled at least one exemplar in the category that possessed the corresponding trait. By contrasting memory for individual category‐attribute pairings with generic judgments, we aimed to discourage participants from interpreting generics as memory tests.

We expected that both question types are sensitive to differentiation. Specifically, we hypothesized that there is a memory advantage for distinctive attributes of the second‐learned category (Woitzel & Alves, 2024) and that the corresponding generics are more likely judged as true (replicating the results from Experiments 1 and 2). However, we expected that both question types are qualitatively different in the sense that recall of category‐attribute pairings is overall stronger than generic acceptance, which would indicate that generic judgments are not interpreted as memory tests. By comparing overall memory performance in Experiment 3 with overall generic acceptance in the equivalent condition of Experiment 2, we were also able to test whether participants interpreted their task as memory tests in the previous experiments.

## 4 Experiment 3

### 4.1 Method

#### 4.1.1 Participants

In line with the preregistered protocol (https://aspredicted.org/XM8_Z1H), we recruited 250 participants via Prolific. One participant was excluded for failing attention check items, following the preregistered criterion. This resulted in a final sample size of 249 (112 women, 130 men, 7 nonbinary; *M*_age_ = 31.8, *SD*_age_ = 9.3).

#### 4.1.2 Design

The experiment employed a 3 (prevalence: 25% vs. 50% vs. 75%) x 2 (distinctiveness: distinctive vs. shared) x 2 (position: first vs. second) x 2 (question: memory test vs. generic sentence) design. All factors were manipulated within‐subjects.

#### 4.1.3 Stimuli, procedure, and dependent variables

Stimuli, procedure, and dependent variables were adopted from Experiment 2 but modified to implement the memory test items. Specifically, we presented an item before each generic sentence that asked participants whether they recalled at least one exemplar in the category that possessed the trait (e.g., “Do you recall at least one Aquarid that reflects incident light?”) to which participants responded with “yes” or “no” by clicking the respective button. Immediately below each of these questions, we presented the text “Would you agree with this general statement”: followed by the corresponding generic sentence (e.g., “Aquarids reflect incident light.”) to which participants also responded with “yes” or “no,” similar as in Experiments 1 and 2. On the previous screen which instructed participants more generally to their task, we exchanged the exact wording to the following: “First, you will be asked whether you remember at least one exemplar that had a certain attribute. You will then be asked to judge a more general statement about the kind. Decide whether you think the sentences are an applicable description of the kind in general.” In addition, since we did not manipulate the factor domain anymore, all participants received instructions that matched with the scenario about sea creatures (rather than stone kinds) throughout the experiment.

### 4.2 Results

**Fig. 3:**
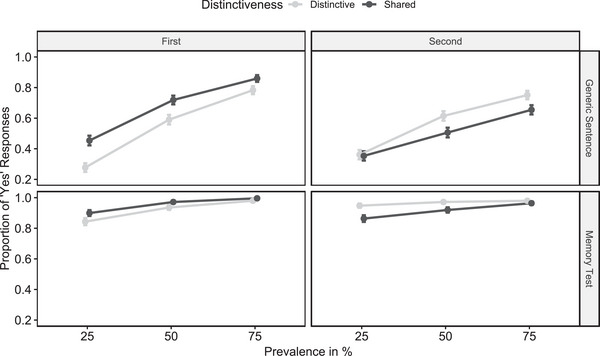
Study 3 results. *Note*. Proportion of accepted generic sentences as a function of prevalence, distinctiveness, position, and question (Study 3). Error bars represent the standard errors of means.

Data and analysis scripts are available on OSF. Descriptive results are summarized in Fig. 3. We used several generalized mixed effects models to analyze the data.^2^ With a first group of models, we aimed to test whether memory for category‐attribute pairings was higher than the acceptance of corresponding generic sentences. We started with a model in which single judgments were predicted by prevalence, distinctiveness, position, and question as well as their interactions as fixed effects, while including participant intercepts as random effects. We observed a significant main effect of question, *z* = 25.12, *p* < .001, indicating that participants more often memorized individual category‐attribute pairings than they accepted the corresponding generic. In another model, we repeated this analysis but used generic judgment data from Experiment 2 (instead of Experiment 3) to compare memory performance and generic acceptance across experiments. Since we only used sea creatures (but not stone kinds) in Experiment 3, we also included Experiment 2 data only from participants who learned about sea creatures. Again, we observed a significant main effect of the factor question, *z* = 3.08, *p* = .002. This indicates that memory performance was higher than the acceptance of corresponding generics, also across experiments.

With a second group of models, we tested whether the distinctiveness advantage for second‐learned categories found in both previous experiments occurred in both generic judgments as well as the memory test. To test generic judgments in isolation, we employed a model in which generic judgments were predicted by prevalence, distinctiveness, and position as well as their interactions as fixed effects, while including participant intercepts as random effects. Replicating the main result of Experiments 1 and 2, we observed a significant interaction between distinctiveness and position, *z* = 6.75, *p* < .001. Contrast tests (two‐tailed) revealed that distinctive (vs. shared) attributes were more likely accepted for the second‐learned category, *z* = 3.46, *p* < .001. In contrast, distinctive (vs. shared) attributes were less likely accepted for the first‐learned category, *z* = 6.02, *p* < .001. We repeated this analysis but excluded all trials in which participants failed to recall the corresponding category‐attribute pairing in the previous memory test. The results were statistically identical, that is, we observed a significant interaction between distinctiveness and position, *z* = 5.73, *p* < .001, with contrast tests revealing an advantage for distinctive attributes in the second‐learned category, *z* = 2.62, *p* = .009, and an advantage for shared attributes in the first‐learned category, *z* = 5.41, *p* < .001. Finally, we employed a model in which we only included responses to the memory test items. Again, we observed a significant interaction between distinctiveness and position, *z* = 4.53, *p* < .001, with contrast tests revealing an advantage for distinctive attributes in the second‐learned category, *z* = 3.73, *p* < .001, and an advantage for shared attributes in the first‐learned category, *z* = 2.85, *p* = .004.

### 4.3 Discussion

In Experiment 3, we observed three major results. First, memory for category‐attribute pairings was stronger than the acceptance of corresponding generic sentences. This pattern also emerged across experiments, that is, memory performance in Experiment 3 was higher than generic acceptance for the same category‐attribute pairings in Experiment 2. This indicates that generic judgments were not solely interpreted as a memory test, neither in this nor in the previous experiments. However, while memory for individual instances cannot explain the full pattern of results, we cannot rule out the possibility that a subset of participants treated the task like a memory test in the previous experiments, potentially inflating overall generic acceptance.

Second, the key result pattern of both previous experiments was replicated. Specifically, generic sentences were more likely judged as true when generalizing distinctive (vs. shared) attributes, but only for the second‐learned category. Importantly, this pattern remained robust even when excluding generic judgments for category‐attribute pairings that were not previously recalled. Thus, while memory for category‐attribute pairings was also sensitive to differentiation (see also Woitzel & Alves, 2024), this does not fully account for the same pattern in the judgment of corresponding generic sentences.

Finally, generic judgments differed from those in the previous experiments in various other respects. Most notably, their overall acceptance was markedly lower, and several factors might explain this difference. As mentioned, it might be possible that a subset—but not all—participants in the previous experiments treated the task like a memory test, leading to overall higher acceptance rates, whereas Experiment 3 explicitly contrasted generic judgments with memory for individual instances. Additionally, the memory items added in Experiment 3 may have prompted participants to systematically search for all instances that possessed the attribute, potentially nudging participants to base the subsequent generic judgment more on prevalence. This might have led participants to accept generics more cautiously, particularly in low‐prevalence conditions. Regardless of the reason for the low acceptance rates in Experiment 3, this finding indicates that generic judgments are sensitive to the current experimental context and influenced by prior judgments, which should be investigated in future research (see also Bless, Strack, & Schwarz, 1993). Overall, the results of Experiment 3 confirm that the distinctiveness effect on generic judgments for second‐learned categories is robust above and beyond memory for individual category‐attribute pairings.

## 5 General discussion

In three experiments, we observed that generic sentences about fictitious categories are more likely accepted when they refer to distinctive attributes, but only for the second‐learned category. This pattern suggests that generic sentences mirror cognitive representations of categories (Gelman, 2010; Leslie, 2008). Specifically, distinctive attributes are prioritized during category learning and subsequently linked more strongly to the category. This only affects representations of second‐learned, but not first‐learned categories since perceivers can recognize which attributes are distinctive for second‐learned, but not first‐learned categories during learning (Alves et al., 2018; Sherman et al., 2009). Apparently, the resulting asymmetries in category representations are also expressed in the acceptance of generic sentences, which confirms the core prediction of a cognitive view on genericity. In contrast, the present results are hardly explicable from either a semantic or a pragmatic perspective. Specifically, semantic rules do not plausibly prescribe that generic sentences have different meanings for first‐ and second‐learned categories. Pragmatic principles might predict that generic sentences are sensitive to the current conversational context, but not to the order of learning about comparison categories.

### 5.1 Implications

The insight that generics about distinctive (vs. shared) attributes are more likely accepted may help explain seemingly paradoxical truth judgments. For instance, “Birds lay eggs” might be considered true because laying eggs distinguishes birds from mammals and is thus strongly linked to birds. In contrast, “Birds are female” might be considered false because being female does not distinguish birds from other animals and is thus not strongly linked to birds. Similar patterns emerge in social generics. For instance, “Germans eat sauerkraut” might be considered true because, on average, more sauerkraut is consumed in Germany than in other countries. In contrast, “Germans are right‐handed” might be considered false because the likelihood of being right‐handed is equally high across countries. Understanding the truth conditions of social generics has major theoretical and practical implications for stereotyping. Previous research suggests that stereotyping of individuals is driven more by generic beliefs than by prevalence estimates. For instance, whether somebody expects a lawyer to be greedy is better predicted by their endorsement of the generic “Lawyers are greedy” than by their estimate of how many lawyers are actually greedy (Hammond & Cimpian, 2017; see also Khemlani et al., 2012). Combining this insight with the present findings suggests novel strategies for reducing stereotyping. Simply correcting inaccurate prevalence estimates (e.g., informing people that only few lawyers are greedy) may be insufficient. Instead, it may be more effective to tackle the generic belief that lawyers are greedy. Specifically, a promising approach might be to challenge the perception of distinctiveness (e.g., informing people that individuals in other professions can also be greedy).

A novel insight from our findings is that the acceptance of generics about distinctive and shared traits is moderated by learning position. Specifically, generics about distinctive attributes are more likely accepted when describing second‐learned, but not first‐learned categories. Are there categories in the social world that can be systematically assigned to being either first‐ or second‐learned? Not always, as most social groups are learned in parallel rather than sequentially. For instance, people likely do not acquire information about lawyers and judges in strictly separated temporal order (unless embarking on a legal career determines a certain learning sequence). In such cases, the acquisition of information about both comparison categories is shaped by differentiation from the other category. Applied to generic sentences, when referring to (allegedly) distinctive attributes of either category (e.g., “Lawyers are greedy” or “Judges are rational”), generics can be expected to be accepted more likely. However, in other cases, clear first‐ and second‐learned groups do exist. Specifically, people typically form impressions of in‐groups and majorities before they form impressions of out‐groups and minorities (Alves et al., 2018; Bergh & Lindskog, 2019). Thus, generic sentences about distinctive traits of out‐groups and minorities might be endorsed more likely than generic sentences about distinctive traits of in‐groups and majorities. This implies that generic sentences are judged differently depending on a perceiver's own group membership. For instance, “Germans eat sauerkraut” might be more likely accepted by non‐Germans than by Germans themselves because non‐Germans experience the distinctiveness of this behavior during their initial learning about Germans. On a more fundamental level, this suggests that generic sentences’ truth conditions are not necessarily shared between people, as different people have different perspectives and learning histories. Again, this challenges formal semantic approaches of genericity that assume objectively valid rules that determine the truth value of a generic sentence.

### 5.2 Limitations

While we believe that our research facilitates the understanding of generic sentences in important aspects, we also wish to emphasize some limitations. First, although the present experiments provide causal evidence for the influence of attribute distinctiveness and learning order on judging generic sentences, we do not claim these variables alone offer a comprehensive explanation of generics. As elaborated throughout this article, we locate our findings in a cognitive framework of generics, which posits that generic sentences express mental representations of categories. These representations are driven not only by differentiation but also by various other cognitive and social factors. For instance, as noted in the Introduction, characteristic attributes that are relevant for causal theories about categories are strongly linked to these categories. Generic sentences about these characteristic attributes are typically judged as true, potentially overriding distinctiveness. For instance, “Dogs reproduce” seems true although reproducing is not distinctive for dogs (in fact, neutering might be more common in dogs than in other animals). Rather than contradicting our findings, this example illustrates another facet of category representation, aligning with the broader cognitive perspective. In other cases, distinctiveness might covary with other stimulus attributes. For example, “Birds lay eggs” and “Lions have manes” generalize attributes that are both distinctive and characteristic. While differentiation provides a parsimonious explanation for the acceptance of these generics, we recognize that other factors also contribute to their perceived truth.

Second, the generalizability of our findings might be limited due to the artificial experimental setup. More specifically, we deliberately selected traits that are not charged with a priori assumptions about their prevalence and causal status. This has the advantage of maximizing internal validity (i.e., allowing an examination of distinctiveness without being confounded with other variables). Nevertheless, generic sentences might become rather invariant against differentiation when other variables enter the equation. For instance, the definitional generic “Aquarids are sea creatures” would likely receive strong agreement although being a sea creature did not distinguish Aquarids and Neptunians in our experimental paradigm. Again, this underlines that distinctiveness influences the acceptance of generic sentences in otherwise neutral conditions but that it can be overridden by other factors.

Finally, the cognitive approach of generic sentences has theoretical limitations because it lacks precise assumptions about the cognitive architecture of category representations. Prominent literature assumes a primitive process of generalizing attributes from instances to categories (Leslie, 2008), but does not specify how category‐attribute links are mentally organized and retrieved. Future work should take this up in more detail to allow more precise predictions about generic judgments. For instance, a comprehensive cognitive theory of generic sentences might address (a) whether category representations are purely explicit or have independent implicit components (e.g., Gawronski, Ledgerwood, & Eastwick, 2022), (b) if and how category representations and their retrieval are malleable by the current context (e.g., Casper, Rothermund, & Wentura, 2010), and (c) how generic beliefs coexist with statistical knowledge about categories (e.g., Bian & Cimpian, 2017).

### 5.3 Conclusion

Generic sentences are puzzling because they are used frequently and effortlessly, yet they are complex to analyze from a semantic perspective. Cognitive approaches of genericity attempt to resolve this puzzle by identifying asymmetries in the mental representation of categories that might be mirrored in seemingly paradoxical generics. We found evidence that generics are more likely considered true when referring to distinctive attributes, but only for categories encountered second, a pattern that reflects learned category representations. Our findings further imply that generic sentences’ truth judgments might be more flexible and less likely shared between people than often assumed. We encourage researchers to further draw on psychological theory to better understand generic sentences and their truth conditions.

## Open science practices

All experiments were preregistered. We report all conditions and all dependent variables in all experiments. In both the preregistrations and the manuscript, we report how we planned the sample sizes and the applied exclusion criteria. Data and analysis scripts for all experiments and preregistrations are available for download from the Open Science Framework: https://osf.io/xuy4n/?view_only=c7065479ffb7487a9002c620ac088648

## Research ethics

Informed consent was obtained from all participants included in the study. All procedures were performed in accordance with the ethical standards of the institution's Human Research Ethics Committee. A formal approval of the Ethics Committee is available.

## Data Availability

Data, analysis scripts, and materials of all experiments are available for download from the Open Science Framework: https://osf.io/xuy4n/?view_only=c7065479ffb7487a9002c620ac088648

## Notes

1 We conducted a simulation‐based power analysis to validate our sample size choice. A description of the procedure as well as the script is available on OSF.

2 The analyses reported here extend the preregistered protocol. We originally aimed to test for a main effect of question type as well as an interaction between distinctiveness and learning order. While we report these analyses here, we also report follow‐up analyses that we did not consider at the time of preregistration.
